# Brain and whole-body FDG-PET in diagnosis, treatment monitoring and long-term follow-up of primary CNS lymphoma

**DOI:** 10.2478/raon-2013-0016

**Published:** 2013-05-21

**Authors:** Sofiane Maza, Ralph Buchert, Winfried Brenner, Dieter Ludwig Munz, Eckhard Thiel, Agnieszka Korfel, Philipp Kiewe

**Affiliations:** 1 Department of Nuclear Medicine, Vivantes MVZ Spandau, Berlin, Germany; 2 Department of Nuclear Medicine, Charité-Universitätsmedizin, Berlin, Germany; 3 Department of Hematology, Charité-Universitätsmedizin, Berlin, Germany

**Keywords:** PET-CT, primary central nervous system lymphoma, response assessment, imaging

## Abstract

**Background:**

Positron emission tomography (PET) with F-18-labeled fluorodeoxyglucose (FDG) provides remarkable accuracy in detection, treatment monitoring and follow-up of systemic malignant lymphoma. Its value in the management of patients with primary central nervous system lymphoma (PCNSL) is less clear.

**Patients and methods:**

In a prospective trial, 42 FDG-PET examinations were performed in ten immunocompetent patients with newly diagnosed or recurrent PCNSL before and repeatedly during and after the treatment. Brain and whole body FDG-PET were compared to brain MRI and extra-cerebral CT, respectively.

**Results:**

Before the treatment, 6 of 10 patients had congruent findings on FDG-PET and MRI of the brain. Three patients had lesions on brain MRI, not detected by FDG-PET. One patient had additional FDG-PET positive lesions inconspicuous in MRI. The follow-up suggested FDG-PET to be false positive in these lesions. After the treatment, brain PET was in agreement with MRI in 6 of 8 patients. In the remaining 2 patients there were persistent lesions in brain MRI whereas FDG-uptake was reduced to normal values. In the long-term follow-up of 5 patients (63–169 weeks), 3 patients retained normal in both PET and MRI. In 2 patients a new focal pathologic FDG-uptake was detected 69 and 52 weeks after the end of the treatment. In one of these patients, recurrence was confirmed by MRI not until 9 weeks after PET.

**Conclusions:**

Brain FDG-PET may contribute valuable information for the management of PCNSL, particularly in the assessment of the treatment response. Integration of FDG-PET into prospective interventional trials is warranted to investigate prognostic and therapeutic implications.

## Introduction

Magnetic resonance imaging (MRI) is the standard diagnostic modality when primary central nervous system lymphoma (PCNSL) is suspected, often showing characteristic radio-morphological features such as lesion location adjacent to cerebrospinal fluid (CSF) space, strong and homogenous contrast-enhancement, moderate oedema and absence of necrosis. MRI of the brain is commonly used for the evaluation of the treatment response, however, with several limitations. Contrast-enhancement reflecting blood-brain barrier (BBB) disruption may be caused not only by the tumour, but also by surgical intervention, radiotherapy or chemotherapy. Misinterpretation as tumour residuum in these latter cases results in the unnecessary treatment which might cause late neurotoxicity. In follow-up, the differentiation of malignant lesions from other lesions like inflammatory processes or scars can sometimes be difficult in MRI.^1^

F-18-labelled fluorodeoxyglucose (FDG) positron emission tomography (PET) has demonstrated remarkable sensitivity in the detection of systemic non-Hodgkin’s lymphoma (NHL). Furthermore, it has been shown to provide high accuracy in the differentiation between cerebral lymphomas and either high-grade gliomas or infectious lesions in patients with acquired immunodeficiency syndrome (AIDS).^1^–^3^ As FDG-uptake in the brain relates to the rate of tissue glucose metabolism, FDG-PET is not limited to the assessment of BBB integrity but provides functional information on tumor viability.^4^

A few studies have evaluated FDG-PET in immunocompetent PCNSL patients.^5^–^13^ However, no study has systematically addressed the value of repeated FDG-PET in both treatment-monitoring and long-term follow-up in PCNSL in the same patients. Therefore, the aim of the present prospective study was to evaluate potential diagnostic benefits of FDG-PET in a sample of immunocompetent PCNSL patients in the assessment of disease extent prior to the treatment as well as in treatment monitoring and in long-term follow-up.

## Patients and methods

The research has complied with all relevant national regulations and institutional policies and has been approved by the authors’ institutional review board or equivalent committee.

### Patients

The study included ten immunocompetent patients (5 females, median age at inclusion 54.5 years) with PCNSL who were scheduled for the treatment at our institution. Seven patients had newly diagnosed PCNSL with a histological proof of high-grade B-cell lymphoma. Systemic lymphoma was excluded by computed tomography (CT) and bone marrow biopsy. Three patients had recurrent PCNSL with a histological proof at first diagnosis. HIV infection was excluded in all patients.

All 7 patients with newly diagnosed PCNSL received chemotherapy with high-dose methotrexate (HDMTX) for up to a total of 6 cycles. Of 3 patients treated for recurrent disease, one patient (No. 3) received HDMTX plus ifosfamide, and 2 patients (No. 9 and 10) were treated with Y-90-labeled (90Y) ibritumomab tiuxetan.

### Imaging protocol

Several imaging sessions were scheduled for each patient, each session including both FDG-PET (brain and whole-body) and MRI (brain only). Initially, a ‘baseline session’ was performed before the treatment initiation, followed by ‘therapy monitoring sessions’ after 3 HDMTX cycles (in the 7 patients with newly diagnosed PCNSL), and 4–6 weeks after the completion of the treatment. Thereafter, ‘follow-up sessions’ were performed every 3–4 months.

### FDG-PET imaging

Brain FDG-PET scans were started 60 min after intravenous injection of about 370 MBq FDG with an acquisition time of 20 minutes, followed by a whole body scan including 6–8 bed positions. An ECAT EXACT system (30 sessions) and later a Biograph 16 system (12 sessions) was used (Siemens, Erlangen, Germany). Transversal images were generated using the standard reconstruction algorithm of the system software. The attenuation correction was based on either a Ge-68 transmission scan (ECAT EXACT) or a low-dose CT (Biograph).

Reconstructed images were first analysed visually. Suspect areas of focally increased FDG uptake were further analysed by region of interest (ROI) analysis. In the brain, circular ROIs of 6.4 mm diameter were placed manually in the lesion, centred at the hottest voxel. The maximum standardized uptake value (SUV max, maximum activity concentration in the ROI / (injected dose / body weight)) was used for semi-quantitative analysis. In addition, an FDG uptake ratio was computed by dividing mean SUV in the ROI by mean SUV in the (manually placed) mirror ROI in the other hemisphere (normal tissue). This scaling procedure reduces variability by elimination of global effects and, thus, improves the power for the detection of local pathological changes.

In order to determine the normal range of the FDG uptake ratio in the present study, the uptake ratio analysis was performed for healthy cortical gray matter, healthy white matter, and healthy thalamus in each brain FDG PET scan in each patient. Univariate analyses of variance with uptake ratio as dependent variable, tissue type (gray, white, thalamus) as fixed factor, and time (delay in days to baseline scan of the same patient) as covariate did not detect any significant effect. Therefore, all uptake ratios were grouped together, resulting in mean ± 1 standard deviation of the sample = 1.010 ± 0.045 (range 0.913 – 1.149). Defining the upper threshold of the normal range by mean + 3 standard deviations resulted in a threshold of 1.15 for the uptake ratio to be pathologically increased.

### MR imaging

All MRI studies were carried out using a standardized protocol including sagittal T1-weighted spin echo (slice thickness 5 mm, inter-slice gap 0.5 mm), axial T2-weighted fast spin echo, coronal FLAIR and axial T1-weighted spin echo before and after Gd-DTPA injection. Additional sagittal and coronal T1-weighted spin echo images were acquired when contrast enhancement was observed on axial slices. The response on MRI was evaluated according to International PCNSL Group (IPCG) criteria.^14^ For response evaluation, patients had to be off-steroids to differentiate between the effect of steroids and chemotherapy.

FDG-PET and MRI were interpreted independently by an experienced nuclear medicine physician and an experienced radiologist, respectively. Both readers were blinded to clinical data.

## Results

### Baseline

**FIGURE 1:**
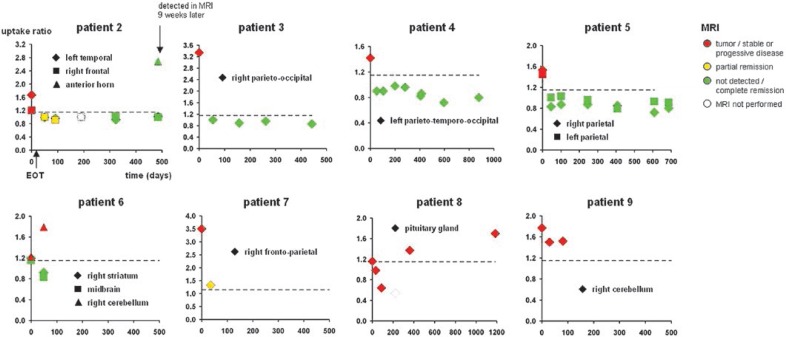
Summary of brain PET and MRI findings in all patients with at least one follow-up session. Patients 1 and 10 did not show any suspect lesion in the FDG-PET of the brain.

The results at baseline are summarized in Table 1 and Figure 1. In the 7 newly diagnosed patients, baseline MRI was performed pre-biopsy in 3 and post-biopsy in 4 patients. Of patients with pre-biopsy MRI (No. 2, 4, and 5), two patients had stereotactic biopsy, the other patient (No. 5) had open resection of one lesion. Of patients with post-biopsy MRI (No. 1, 6, 7, and 8), one patient (No. 1) had stereotactic biopsy, one patient (No. 8) open biopsy, and 2 patients (No. 6 and 7) had open lesion resection.

FDG-PET was performed after MRI and post-biopsy in all patients. Seven patients were on corticosteroids at the time of the PET examination.

Number and location of cerebral lesions at baseline was in agreement between PET and MRI in 6 of 10 patients (No. 3, 4, 5, 7, 8, 9; the right frontal MRI lesion in patient No. 5 had been resected prior to FDG-PET and, therefore, was excluded from the analysis (Table 1). Disagreement between PET and MRI at baseline occurred in 4 of 10 patients (No. 1, 2, 6, 10). In 3 of these patients (No. 1, 2, 10) FDG-PET of the brain showed less lesions than MRI. In 2 of these 3 patients (No. 1 and 10) FDG-PET was rated entirely normal, whereas MRI detected a single contrast enhancing lesion suspicious of PCNSL in the left thalamus (No. 1) and multiple lesions in the cerebellum (No. 10), respectively. In the 3^rd^ of these patients (No. 2) FDG-PET was negative in a MRI lesion in the left cerebellum, but positive in the 2 further MRI lesions of this patient. In the remaining patient with disagreement between FDG-PET and MRI at baseline (No. 6), FDG-PET showed 2 lesions (right striatum, midbrain) which had not been detected by MRI. A lesion in the right cerebellum of this patient was detected by both modalities. Maximal SUV value of brain lesions in the PET ranged between 3.6 and 12.5 (median 5.3), the uptake ratio ranged between 1.2 and 3.5 (median 1.5).

Whole-body FDG-PET detected extra-cerebral lesions in 2 of 10 patients (No. 3 and 4). In patient No. 3, who had recurrent PCNSL, pathologic FDG-uptake was detected in the lung, mediastinal lymph nodes and thoracic wall. These findings were confirmed as asymptomatic systemic lymphoma manifestations by subsequent CT. In patient No. 4, pathologically increased FDG-uptake was found in the left kidney without a correlative on CT. The lesion was confirmed in the follow-up PETs.

### Therapy monitoring

The results of the therapy monitoring are summarized in Table 2 and Figure 1. Imaging for therapy monitoring was performed after the completion of the therapy in all cases. An additional imaging session during 3 cycles of HDMTX was performed in 1 patient (No. 3). All patients were off steroids at the time of monitoring sessions.

The evaluation of the therapy response by MRI was performed in 9 patients. Of these, complete response (CR) was found in 3 patients, partial response (PR) in 2, stable disease / minimal response (SD/MR) in 2, and progressive disease (PD) in 2 patients.

**FIGURE 2:**
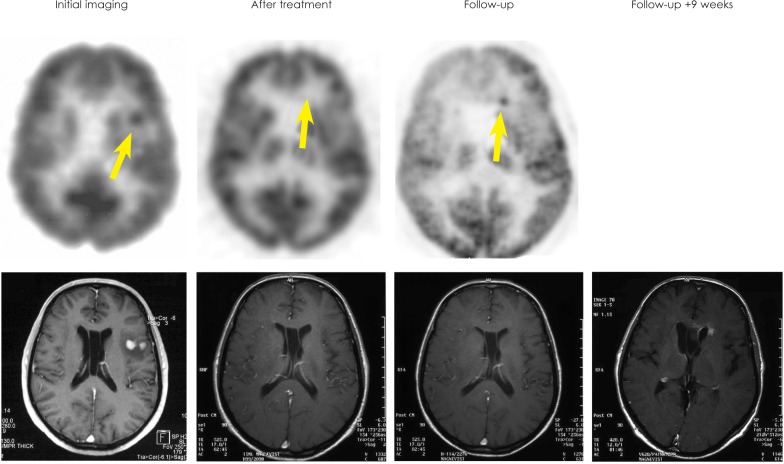
Baseline PET and MRI of patient No. 2 shows left temporal lesion. No pathological FDG-uptake was observed after therapy but a new lesion in the anterior horn was seen on PET 69 weeks later (SUV=10.5). MRI at this time point was still categorized as complete remission. A contrast-enhancing lesion appeared on MRI 9 weeks thereafter.

Therapy monitoring by FDG-PET was performed in 8 patients. FDG-PET was in agreement with MRI in 6 of these: CR in MRI and normal FDG-PET in 3 patients (No. 3, 4, 5), PR in MRI and decreased FDG uptake compared to baseline (but still elevated) in 1 patient (No. 7), stable disease in MRI and persistent pathologic FDG uptake in 1 patient (No. 9), and PD in MRI and increase of pathologic FDG uptake in 1 lesion in PET in 1 patient (No. 6). There was a disagreement between MRI and FDG-PET in the remaining 2 patients. FDG-PET was rated normal in both of these patients, whereas MRI was rated as PR (No. 2) or even SD/ MR (No. 8). The first of these patients (No. 2) did not show recurrence of the lesions detected at baseline during the follow-up of 69 weeks, suggesting that the normal FDG-PET finding after the therapy was true negative, despite of the disease progression with a novel lesion in another part of the brain at this late time point (frontal horn, Figure 2). In the second of these patients (No. 8), recurrence was observed in FDG-PET 52 weeks after the treatment.

### Follow-up

Results of the follow-up are summarized in Table 3 and Figure 1. A long-term follow-up by both MRI and FDG-PET was performed in 5 patients (No. 2, 3, 4, 5, 8). Median time of the follow-up was 98 weeks (range 63–169). In 3 of these patients (No. 3, 4, 5) both MRI and FDG-PET showed a complete remission during the whole period of the follow-up. In 1 patient (No. 2), a new pathologic FDG uptake (SUV max = 10.5, uptake ratio = 2.7) was detected by PET in the anterior horn after 69 weeks. MRI revealed tumour r relapse not until 9 weeks after positive PET (Figure 2). In another patient (No. 8), a new pathologic FDG uptake (SUV max = 8.6; uptake ratio = 1.4) was seen on PET after 52 weeks along with documented stable disease on MRI.

## Discussion

First results on FDG-PET in PCNSL were reported by Rosenfeld *et al.* who, investigating 10 patients, found FDG-uptake in PCNSL lesions to be similar to that of anaplastic gliomas.^10^ In a series of seven patients evaluated at the time of initial diagnosis, Palmedo *et al.* found a good correlation between gadolinium enhancement in MRI and focal FDG-uptake on PET with merely one lesion missed by PET and another lesion visible only on the FLAIR MRI sequence.^9^ In the present study, there was an agreement between PET and MRI before therapy in 6 of 10 patients. In 3 of the remaining 4 patients, FDG-PET showed fewer lesions suspicious of PCNSL than contrast-enhanced MRI. Two of these 3 patients were rated normal (no lesion) in FDG PET. This discrepancy might be explained by initiation of corticosteroid therapy between MRI and PET. Corticosteroid-induced reduction of FDG-uptake has been reported in cerebral lymphoma.^5^ In one patient, the residual contrast enhancement of a cerebellar lesion on MRI might have been caused by postoperative BBB dysfunction. In this case the negative FDG-PET might indicate the absence of vital tumour after the resection. In 1 patient, PET showed brain lesions of the increased FDG uptake that were not detected in MRI. However, neither specific neurological symptoms nor follow-up supported the presence of lymphoma in these lesions. Most likely the increased tracer uptake was due to benign postoperative processes in these cases. In summary, FDG-PET might provide valuable information in addition to MRI with respect to disease extent at initial imaging before therapy in most patients.

In the present study, FDG-PET included a whole-body scan in addition to the brain scan, in contrast to most previous studies. Whole-body FDG-PET was in agreement with CT in all patients newly diagnosed with PCNSL. The increased FDG-uptake in the left kidney in one newly diagnosed patient was without correlate in CT, also in retrospective inspection of the CT, and did not change in follow-up PET, and, therefore, most likely was not malignant. Pathological extra cerebral FDG-uptake was found in one patient with PCNSL relapse, and was subsequently confirmed as systemic lymphoma by CT. Based on this finding, the treatment was changed in this patient: systemic chemotherapy was initiated instead of whole-body immunoradiotherapy as had been planned prior to whole-body PET. This important finding might have been missed in clinical routine patient care, since abdominal or thoracic CT is not routinely performed in relapsed PCNSL patients. A previous case report by Karantanis *et al.* also suggests that whole-body PET might be superior to CT in the detection of extracranial disease in PCNSL.^12^ In a retrospective study, Mohile *et al.* evaluated the contribution of whole-body FDG-PET in staging and restaging of PCNSL patients.^6^ These authors found systemic disease in 7% of patients with suspected PCNSL, which would have been missed with conventional CT. This rate was even higher (27%) in patients with CNS relapse. Thus, whole-body FDG-PET might result in change of the treatment strategy in a considerable fraction of patients.

Response evaluation can be difficult in PCNSL, since the residual contrast enhancement caused by the treatment-induced disruption of the BBB is not infrequent. The differentiation between CR and PR, which is of paramount importance for the decision about the further treatment, is often particularly difficult. Standardized criteria for the evaluation of the treatment response in PCNSL include the category of ‘unconfirmed complete remission’ (CRu) for patients needing corticosteroids despite disappearance of all gadolinium-enhancing lesions on MRI and patients with a small but persistent contrast enhancement after biopsy or focal haemorrhage.^14^ FDG-PET is expected to be particularly useful in these cases. In the present longitudinal prospective study, FDG PET was performed for the response evaluation as well as during the follow-up in the same patients. FDG-PET provided different information about the therapy response than MRI in 2 of 8 patients. In both patients, FDG-PET indicated CR despite of the persistent contrast enhancement in MRI. There was no recurrence of the initial lesion during the follow up of 69 weeks in one of these patients, suggesting a true negative FDG-PET, despite of disease progression with novel lesions in other parts of the brain. In the second of these patients, FDG-PET indicated a complete remission whereas MRI findings were categorized as stable disease / minor response. The recurrence was observed in FDG-PET only 52 weeks after the treatment. These findings are in agreement with the results of Palmedo *et al.*, who reported truly negative PET findings (confirmed by follow-up) despite the persistence of lesions on MRI in three of six patients.^9^ A response to initial chemotherapy as assessed by MRI has consistently been associated with the prolonged survival in PCNSL.^15^,^16^ The present findings suggest that FDG-PET might improve the response assessment and, therefore, might provide improved prognostic power compared to MRI. However, further studies with larger patient samples are required to test this hypothesis.

In the long-term follow-up of one patient, relapse was detected by FDG-PET but not by MRI at this time point. Relapse was confirmed by MRI 9 weeks later. However, while FDG-PET might have the potential to detect relapse earlier than MRI, this benefit might not be relevant in clinical routine, because making use of this benefit would require very short intervals between follow-up imaging which cannot be justified on a routine base due to the radiation exposure. In most cases, the relapse is detected by the occurrence of new neurologic symptoms rather than by routine imaging.

The main limitations of the present study are relatively small sample size, heterogeneity among the patients with respect to various factors (newly diagnosed versus recurrence, treatment strategy), a time interval of more than 2 weeks between MRI and PET at baseline in 3 out of 10 patients, corticosteroid treatment at the time of baseline FDG PET in 7 out of 10 patients, use of two different PET scanners with slightly different spatial resolution, and incomplete follow-up in some patients. Nevertheless, the data suggest that FDG-PET may contribute valuable information in PCNSL. Whole-body FDG-PET might be useful for staging prior to therapy. FDG-PET of the brain might be useful for the evaluation of the treatment response, particularly in case of mild residual contrast-enhancement in MRI.

## Figures and Tables

**TABLE 1:** Imaging findings at baseline

**No.**	**First diagnosis (F) / recurrent disease (R)**	**# of CNS lesions on MRI**	**Location and size of lesions on MRI**	**MRI pre/post biopsy**	**Extent of biopsy**	**Steroid treatment**	**Interval between MRI and PET (days)**	**# of CNS lesions on PET**	**Location of pathologically increased focal FDG uptake according to visual analysis**	**SUVmax**	**Uptake ratio**	**Whole-body PET**
**1**	F	1	left thalamus	post	Stereotactic	yes	14	0				normal
**2**	F	3	left temporal (2.2×1.25cm)	pre	Stereotactic (left temporal lesion)	yes	14	2	left temporal	4.1	1.7	normal
			right frontal						right frontal	4.2	1.2	
			left cerebellum									
**3**	R	1	right parieto-occipital (4×2cm)	NA	No biopsy	no	7	1	right parieto-occipital	9.7	3.3	Pathologically increased FDG-uptake in lung, thoracic wall and mediastinal lymph nodes (n=11)
**4**	F	1	left parieto-temporo-occipital (3×3cm)	pre	Stereotactic	yes	14	1	left parieto-temporo-occipital	4.2	1.4	Pathologically increased FDG-uptake in left kidney/adrenal gland (stable in follow-up PET)
**5**	F	3	right frontal (3×1.8cm)	pre	Open resection of right frontal lesion	yes	7	2	low FDG-Uptake after open resection right frontal			normal
			left parietal (2×1.4cm)						left parietal	10.7	1.5	
			right parietal (2cm×1.5)						right parietal	12.5	1.5	
**6**	F	1	residual contrast enhancement in right cerebellum (postoperative)	post	Open resection of right cerebellar lesion	yes	28	3	right cerebellum	4.3	1.2	normal
									right striatum	6.0	1.2	
									midbrain	4.6	1.2	
**7**	F	1	right fronto-parietal (4.5×2.5cm)	post	Open resection	yes	21	1	right fronto-parietal	3.6	3.5	normal
**8**	F	1	pituitary gland (1×1cm)	post	Open biopsy	no	10	1	pituitary gland	8.7	1.2	normal
**9**	R	1	right cerebellum (3×2cm)	NA	No biopsy	no	7	1	right cerebellum	10.0	1.8	normal
**10**	R	mult	left cerebellum (3.75×2cm)	NA	No biopsy	yes	23	0				normal

CNS = central nervous system; MRI = magnetic resonance imaging; SUVmax = maximal standard uptake value; TU = tumour; PET = positron emission tomography; NA = not applicable; FDG = F-18-fluorodeoxyglucose; mult = multiple

**TABLE 2:** Evaluation of treatment response

**No.**	**Treatment**	**MRI**	**PET**
1	1× HDMTX	ND (death)	ND (death)
2	6× HDMTX	PR	No pathologic uptake
3	6× HDMTX + Ifosfamide	CR (cerebral) after 3 cycles, CR (thoracic) after 6 cycles	No pathologic uptake in the brain after 3 cycles, No pathologic uptake in the chest after 6 cycles
4	5× HDMTX	CR	No pathologic uptake
5	6× HDMTX	CR	No pathologic uptake
6	2× HDMTX	PD	Increase of pathologic uptake in 1 of 3 lesions
7	2× HDMTX	PR	Pathologic uptake reduced
8	6× HDMTX	SD/MR	No pathologic uptake
9	Ibritumomab tiuxetan	SD	Persistant pathologic uptake
10	Ibritumomab tiuxetan	PD	ND

PET = positron emission tomography; HDMTX = high-dose methotrexate; ND = not done; PR = partial response; CR = complete response; PD = progressive disease; SD = stable disease; MR = minimal response

**TABLE 3:** Long-term follow-up

**No.**	**MRI**	**PET**
1	ND (death)	ND (death)
2	CR at 69 weeks after treatment. Relapse detected 78 weeks after treatment	new pathologic uptake 69 weeks after treatment
3	CR	normal
4	CR	normal
5	CR	normal
6	ND	ND
7	ND	ND
8	PR	Recurrence of pathologic FDG 52 weeks after treatment
9	ND	ND
10	ND	ND

PET = positron emission tomography; ND = not done; PR = partial response; CR = complete response
